# Dose Escalation Trial of Desulfated Heparin (ODSH) in Septic Peritonitis

**DOI:** 10.3389/fvets.2022.862308

**Published:** 2022-04-13

**Authors:** Katie D. Mauro, Michele P. Lambert, M. Anna Kowalska, Vincent J. Thawley, Mortimer Poncz, Cynthia M. Otto

**Affiliations:** ^1^Matthew J. Ryan Hospital, Department of Clinical Science and Advanced Medicine, University of Pennsylvania, Philadelphia, PA, United States; ^2^Division of Hematology, Children's Hospital of Philadelphia, Philadelphia, PA, United States

**Keywords:** canine, heparin, platelet factor 4, sepsis, ODSH

## Abstract

**Objective:**

Septic peritonitis is associated with significant morbidity and mortality. As a potential therapeutic agent in the treatment of sepsis, 2-O, 3-O desulfated heparin (ODSH) reduces histones and platelet factor 4 (PF4) in mouse sepsis models. This pilot clinical trial evaluated the safety and effect of ODSH in client-owned dogs with septic peritonitis.

**Interventions:**

In an IACUC-approved, open-label, prospective, dose-escalation clinical trial in 6 dogs with spontaneous septic peritonitis, ODSH administration was initiated following surgical explore to achieve source control. Acute patient physiology and laboratory evaluation (APPLE_fast_ and APPLE_full_) scores on admission, source of septic peritonitis, requirement for vasopressors, the administration of blood products, and survival to discharge were recorded. Platelet count, cell free DNA (cfDNA) concentration, and platelet factor 4 (PF4) concentrations were measured at the time of each ODSH dosage. A dose of ODSH was administered every 8 hs for a total of 4 doses (maximum total dosage 75 mg/kg) based on a pre-determined escalation protocol. Patients were monitored in the ICU following administration for evidence of clinical hemorrhage.

**Main Results:**

The mean APPLE_fast_ and APPLE_full_ scores on admission were 22 +/- 6 and 32 +/-10, respectively. Four dogs received 4 total dosages of ODSH and 2 dogs received 3 total dosages of ODSH intravenously. The mean total dosage of ODSH administered during the study period was 48.3 +/- 21.6 mg/kg. No dog required dose de-escalation or had any evidence of bleeding. Four dogs survived to discharge.

**Conclusions:**

No adverse effects of ODSH administration were documented in dogs with septic peritonitis. A randomized controlled trial is necessary to evaluate ODSH as a novel therapeutic in the treatment of septic peritonitis.

## Introduction

Sepsis is the result of an overwhelming host response to infection that causes activation of pro and anti-inflammatory cascades (1), and can occur in dogs with septic peritonitis. Sepsis is a significant cause of morbidity and mortality and can result in multi-organ failure (1). While challenging, there is an ongoing need for the development of novel therapeutics in the treatment of sepsis (2).

In sepsis, numerous cytokines, chemokines, and particles participate in the immune response. NETosis, the release of web like structures containing DNA and proteins from activated neutrophils, leads to the release of cell free DNA (cfDNA) and cytotoxic histones that activated platelets and cause endothelial cell injury (3, 4). Circulating histones and PF4 released by activated platelets cause suppression in the production of endogenous anti-coagulants, like activated protein C (aPC) (5). Normally, the production of aPC follows a bell-shaped curve and may be enhanced or limited by the interaction with PF4 (5). Negatively charged heparans bind to PF4 to shift aPC generation along the bell-shaped curve and enhance production (5). Anti-coagulants have been investigated as potential novel therapeutics. Unfortunately, while drugs such as unfractionated heparin may reduce the inflammatory response, their potent anti-coagulant properties create a high risk for hemorrhage, and therefore limits their therapeutic application.

Compared to unfractionated heparin, ODSH binds well to PF4 but with approximately 60-fold less antithrombin activity, thereby extending the potential therapeutic window (5, 6). In murine (mouse) studies, administration of ODSH^1^ can enhance activated protein C (aPC) generation without prolonging the activated partial thromboplastin time (aPTT) and potentially limit thrombosis and help maintain vascular integrity (5). In a research setting, ODSH has been safely administered to healthy dogs (7).

In this open label, dose escalation clinical trial, the safety and biologic effect of ODSH in client-owned dogs with naturally occurring septic peritonitis was evaluated. The primary objective of this study was to evaluate the safety of ODSH^1^ administration in dogs with sepsis. We hypothesized that ODSH administration to dogs with sepsis would not result in bleeding diatheses. We also hypothesized that ODSH administration in this population of dogs would result in a reduction in free PF4 concentration, and a reduction in cfDNA concentration in surviving dogs.

## Materials and Methods

### Data Collection

This IACUC approved (protocol 805939), open label, prospective dose escalation clinical trial was conducted in six dogs, treated at a large veterinary tertiary referral ICU, with spontaneously occurring septic peritonitis. Dogs were considered for inclusion if they had a diagnosis of septic peritonitis and owner signed consent for enrollment. Dogs were excluded if they weighed <5 kg, >50 kg, or if surgical intervention for septic peritonitis was not pursued. Primary outcomes evaluated included adverse events following ODSH administration, dose escalation, and the requirement for dose de-escalation. Secondary outcomes evaluated included platelet count, plasma cfDNA and PF4 concentrations. Demographic data, including age, sex, reproductive status, weight, and breed, were recorded at the time of enrollment. Additionally, APPLE_full_, and APPLE_fast_ scores were performed on all dogs at the time of enrollment (8). The results of intra-operative exploration, biopsy, or culture were recorded as the cause of septic peritonitis. The requirement for transfusion with blood products or administration of vasopressors was recorded.

### Sample Collection and Handling

Blood samples were collected at the time of enrollment (admission to hospital or septic peritonitis diagnosis), immediately post-operatively, and every 8 h in the post-operative period (0–24 h). Samples were handled identically at all time points. Blood was collected via an indwelling catheter (peripheral venous or arterial) or non-traumatic direct saphenous, cephalic, or jugular venipuncture. When blood was collected from indwelling catheters, a discard sample of 6 mL was removed prior to acquisition of the sample. At the time of blood collection, approximately 1.9 mL of blood was obtained and divided between K2 EDTA capillary blood collection tube (0.1 mL) and one tube containing 3.2% sodium citrate (1.8 mL). The tube containing K2 EDTA was stored at 4°C until an automated platelet and white blood cell count were performed). The 1.8mL sodium citrate tube was maintained at room temperature for 15 min prior to aPTT analysis. The established testing protocol and reference interval (72–102 s) for the IDEXX Coag Dx^2^ was utilized to perform all aPTT analyses. Following aPTT analysis, 1 mL of blood was placed into an Eppendorf tube benzamidine (final concentration 50 mM) and centrifuged for 10 min. The supernatant (citrated plasma) was immediately separated into Eppendorf tubes and stored at −20°C until submission to the Poncz research laboratory where it was stored at −80°C until batch analysis of cfDNA and PF4 within a month of receipt. The PF4 concentration was determined using a commercially available Canine Platelet Factor 4 ELISA Kit^3^ at three dilutions (1:500, 1:5,000, and 1:25,000) to achieve a concentration within the standard curve (0–25ng/mL) according to manufacturer instructions. In addition, using western blot analysis, a canine protein of the appropriate size cross reacted with an antibody to human PF4. The concentration of cfDNA, was quantitated using a DNA-specific fluorophore. Specifically, 50 ul of plasma diluted 1:10 in phosphate buffered saline (PBS) was incubated with 50 ul of SYTOX^4^ (1:500 in PBS) for 15 min in the dark in a 96 well plate. The fluorescence was read at 515 lambda absorption and 527 lambda emission. A standard curve was generated using calf thymus DNA (*R*^2^ = −0.9998). All samples were assayed in duplicate. Canine plasma from a healthy dog was included as a control (9).

### ODSH Administration and Monitoring

**Figure 1:**
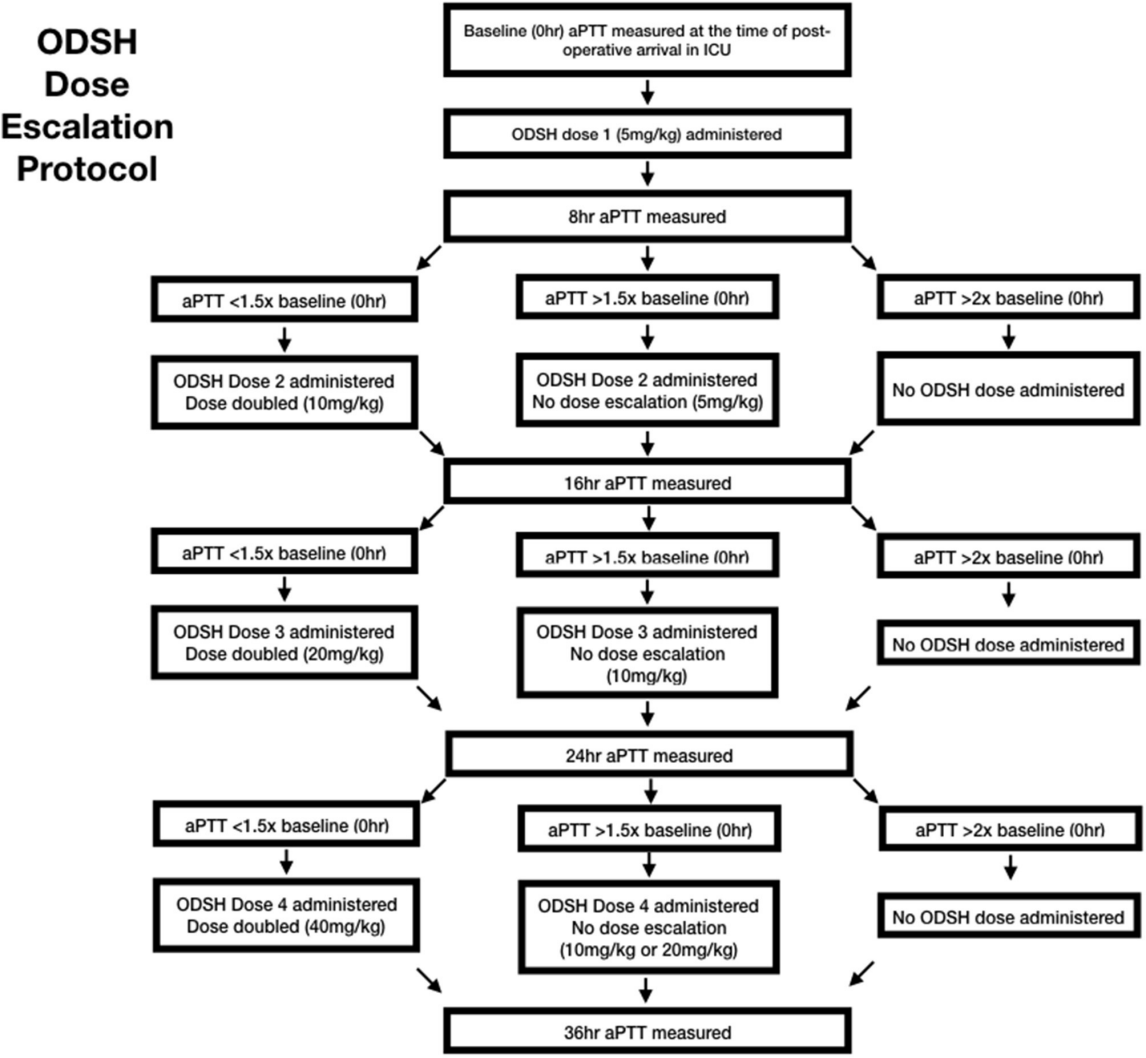
ODSH dose escalation protocol.

The ODSH was diluted with sterile 0.9% saline^5^ to a concentration of 50 mg/mL. The first dose was administered immediately post operatively following clinician investigator examination in the ICU. Doses were administered at the hours 0, 8, 16, and 24. A maximum of four doses were administered, following the dose escalation protocol (Figure 1). At each dosing interval, the aPTT results were used to calculate the next administered dose based on the escalation protocol chart (Table 1). Additionally, the dogs were examined by a clinician investigator and a temperature, pulse rate and respiratory rate were obtained. Each dose was administered as a continuous rate infusion over a period of 5 min. Dogs were monitored for immediate adverse events, including a change in vital parameters or pain associated with administration, by a clinician investigator throughout the dose administration. Immediate adverse events were recorded on a data collection sheet. Throughout the study period, dogs were hospitalized in the ICU and observed for evidence of clinical hemorrhage. While in the ICU, dogs regularly monitored for free fluid accumulation on ultrasound, petechiation, ecchymoses, hematemesis, hematochezia, hematuria, or melena. A daily complete physical examination was also performed by a clinician investigator.

**Table 1:** This is the dose calculation chart used to calculate dose escalation based on measured aPTT.

**Previous dose administered**	**5 mg/kg**	**10 mg/kg**	**20 mg/kg**
aPTT <1.5x Baseline	10 mg/kg	20 mg/kg	40 mg/kg
aPTT >1.5x Baseline	5 m/kg	10 mg/kg	20 mg/kg
aPTT >2x Baseline	0 mg/kg	0 mg/kg	0 mg/kg

### Patient Follow-Up

Survival was defined as live discharge from the hospital. In the surviving dogs, a follow up examination was performed 2 weeks following discharge from the hospital and a follow up phone call was performed at 6 months. In the dogs that died or were euthanized due to clinical deterioration, post-mortem examination was encouraged.

### Statistical Methods

Due to the small number of dogs enrolled, only descriptive statistics are presented. The data was visually inspected and tested for normality using the Shapiro-Wilk test^6^. All normally distributed data was reported as the mean +/- the standard deviation. Any nonparametric data was reported as the median and interquartile range (25, 75%).

## Results

Six dogs (1 female spayed, 1 female intact, 4 male castrated) met the inclusion criteria and were enrolled. The mean weight was 22.2 +/−14.6 kg (range 8.9–46.0 kg). The mean age was 7.6 +/−4.1 years. The mean APPLE_fast_ and APPLE_full_ scores on admission were 22 +/- 6 and 32 +/-10, respectively. The mean aPTT at the time of enrollment was 109.8 +/−25.8 seconds. The source of sepsis identified at the time of surgery was gastrointestinal in three dogs, hepatobiliary and splenic (abscess) in two dogs, and urogenital in one dog. None of the patients had abdominal drains placed post-operatively. All dogs received vasopressor therapy while under anesthesia. Two dogs required vasopressor therapy while in ICU (one survivor, one non-survivor). Five dogs received blood products for oncotic support during their ICU hospitalization (four survivors, one non-survivor). Four dogs received fresh frozen plasma (10–15 mL/kg) and one dog received human serum albumin^7^ (survivor). Four dogs survived to discharge. The two dogs that did not survive were euthanized approximately 24 h after surgery. One dog was euthanized due to owner concerns regarding prognosis and quality of life following the finding of suspected metastatic neoplasia during surgery. Surgical biopsies performed revealed metastatic (spleen and hepatic) sarcoma. One dog was euthanized due to vasopressor resistant hypotension in the post-operative period. Diagnostic necropsies were offered and declined in both dogs. Three dogs were alive at the time of 6-month follow-up.

Following surgical intervention and recovery from anesthesia, all dogs received the initial dose of ODSH (Table 2). The mean total 24-h dosage of ODSH was 48.3 +/−21.6 mg/kg (range 25–75 mg/kg). One non-survivor was escalated at every time point but was euthanized prior to the final (24 h) dose. This dog received a total cumulative dose of 35 mg/kg. The second non-survivor was not escalated above 10 mg/kg and received a total cumulative dose of 25 mg/kg. No dogs required dose de-escalation based on aPTT. Evidence of clinical bleeding, previously defined as free fluid accumulation on ultrasound, petechiation, ecchymoses, hematemesis, hematochezia, or melena, was not documented in any of the dogs.

**Table 2:** Activated partial thromboplastin time measured and ODSH dose administered at baseline and each study time point.


**aPTT (s)**	**Baseline**	**0 h**	**8 h**	**16 h**	**24 h**	**ODSH Dose (mg/kg)**	**0 h**	**8 h**	**16 h**	**24 h**
Dog 1	72	81	88	87	78	Dog 1	5	10	20	40
Dog 2	107	131	149	138	137	Dog 2	5	10	20	40
Dog 3	119	119	75	115	Deceased	Dog 3	5	10	20	Deceased
Dog 4	149	166	182	178		Dog 4	5	10	10	
Dog 5	95	109	118	147	121	Dog 5	5	10	10	20
Dog 6	117	117	150	152	115	Dog 6	5	10	10	10

*Serial measured aPTT and administered ODSH dosages*.

Table 3 displays serial data for white blood cell count, platelet count, PF4 and cfDNA in both surviving and non-surviving dogs. Platelet count was persistently decreased in one of the non-surviving dogs. White blood cell count and measured PF4 concentrations were similar among all dogs. The mean PF4 was 73.8 +/−11.5 ug/ml. The median cfDNA in the patient samples was 0.8 ug/ml (IQR 0.5, 1.5). The control canine plasma cfDNA was 0.5 ug/ml. The two non-surviving dogs had the highest cfDNA concentrations. In these dogs, cfDNA concentrations remained increased at the time of final dose administration.

**Table 3:** This table contains APPLE scores performed at admission and WBC count, platelet count, cfDNA concentration and PF4 concentration collected at each study time point.


	**APPLE_**FULL**_**	**APPLE_**FAST**_**	**WBC (K/μL)**	**Platelet (K/μL)**	**cfDNA (ug/mL)**	**PF4 (μg/mL)**
**Dog 1**	**31**	**16**				
Baseline			18	340	0.9	81.7
0 h			18	340	0.6	104.5
8 h			27.7	289	1.9	77.8
16 h			26.9	293	1.1	87.7
24 h			25.1	253	0.8	97.3
**Dog 2**	**22**	**15**				
Baseline			6.2	530	^**^	^**^
0 h			6.2	530	0.6	56.1
8 h			9.1	212	0.5	79
16 h			14	216	0.5	56.1
24 h			17.3	217	0.6	71.9
**Dog 3**	**36**	**26**				
Baseline			21.8	68	1.5	80.5
0 h			21.8	68	1.4	67
8 h			30.8	79	2.1	57.9
16 h			32.4	147	2.1	60.7
24 h			^**^	^**^	^**^	^**^
**Dog 4**	**42**	**27**				
Baseline			^**^	43	0.5	^**^
0 h			^**^	^**^	1.1	68.5
1.6			^**^	^**^	1.6	74
16 h			70.4	^**^	5.8	70.9
24 h			^**^	^**^	^**^	^**^
**Dog 5**	**18**	**22**				
Baseline			12.8	246	0.4	68.4
0 h			26	267	0.4	68.4
8 h			11.2	341	0.5	67.6
16 h			9.3	237	0.5	71.7
24 h			14.8	207	0.5	80.3
**Dog 6**	**44**	**28**				
Baseline			33.3	249	0.6	72.5
0 h			^**^	^**^	0.9	77
8 h			^**^	^**^	0.8	70.5
16 h			43.7	237	1.7	84.3
24 h			42.5	272	0.7	67.6

Missing data is noted with ^**^. Patient data: apple scores, serial abbreviated platelet count, white blood cell count, cfDNA, and PF4.

## Discussion

This prospective dose escalation trial evaluated the safety of ODSH as a potential novel therapeutic in dogs with naturally occurring septic peritonitis. We aimed to evaluate the safety of ODSH^1^ administration in this population of dogs. In this study, we monitored for hemorrhagic events and hypocoaguability (based on aPTT measurement) following ODSH administration. No hemorrhagic events were documented during the study period. Prolongation of the aPTT (>1.5x baseline) prevented dose escalation in three dogs (one non-survivor and two survivors). None of the dogs required dose de-escalation, and no dogs had clinical evidence of bleeding. Blood products were administered to 5/6 dogs in this study. The administration of plasma products could have altered the aPTT measurements and prevented clinical bleeding. It is also possible that plasma products altered cfDNA and PF4 concentrations in these dogs.

In this study, thrombocytopenia was identified in both non-surviving dogs. Thrombocytopenia is a predictor of mortality in human patients in the ICU (10). Alterations in platelet count have also been reported in dogs with septic peritonitis, however, in one canine study, platelet count was not useful in distinguishing survivors and non-survivors (11). In this study, measured PF4 concentrations were similar among all dogs. There are no studies of PF4 concentrations in septic dogs. The cfDNA concentrations were highest in the two non-surviving dogs. Although, a different fluorphore can lead to absolute differences in the amount of cfDNA measured, the measured concentrations of cfDNA were consistent with what has previously been reported in septic dogs (12) and in a mouse model of sepsis (9). The DNA fluorophore used in this study, was different than that used in other canine studies and may reflect different binding affinity and lower absolute values. We postulate that if higher doses of ODSH were introduced earlier in the course of treatment, these dogs may have responded with decreased cfDNA production, decreased histone release and a better outcome. The use of higher doses of ODSH and the effect on cfDNA production could be evaluated in future studies. A larger study population may have been able to identify a significant difference in PF4 or homologous proteins and cfDNA concentrations among survivors and non-survivors.

This study has several limitations including the small sample size, lack of treatment standardization, the administration of plasma products, and lack of randomization. Additionally, aPC was not measured in this study. Future studies should include a larger study population and a randomized treatment group with placebo control. In this study, ICU care was not standardized, and care was directed at the discretion of the supersizing clinician. Future studies could attempt to standardize care including antimicrobial administration, blood product administration and fluid therapy based on predetermined guidelines. Measurement of aPC and other PF4 related proteins in future studies could aid in the interpretation of the role of canine platelet basic protein in canine sepsis.

This was a pilot study evaluating the safety of ODSH^A^ administration to client-owned dogs with septic peritonitis. Larger studies are necessary to further evaluate the safety of ODSH administration in dogs with septic peritonitis and additional studies are necessary to determine the optimal dose, dosing schedule and evaluate the effect on patient outcomes.

## Data Availability Statement

The original contributions presented in the study are included in the article/supplementary material, further inquiries can be directed to the corresponding author/s.

## Ethics Statement

The animal study was reviewed and approved by Department of Clinical Sciences and Advanced Medicine, University of Pennsylvania. Written informed consent was obtained from the owners for the participation of their animals in this study.

## Author Contributions

KM participated in drafting the original manuscript. All other authors participated in concept, design, analysis, interpretation, and critical revisions. All authors contributed to the article and approved the submitted version.

## Conflict of Interest

The authors declare that the research was conducted in the absence of any commercial or financial relationships that could be construed as a potential conflict of interest.

## Publisher's Note

All claims expressed in this article are solely those of the authors and do not necessarily represent those of their affiliated organizations, or those of the publisher, the editors and the reviewers. Any product that may be evaluated in this article, or claim that may be made by its manufacturer, is not guaranteed or endorsed by the publisher.

## Footnotes

^1^Desulfated 2-O, 3-O heparin (ODSH), ParinGenix Inc, Research Corporation Technologies, Tucson, AZ.

^2^Coag Dx Analyzer, IDEXX, Westbrook, Maine.

^3^Canine PF4 Elisa Kit (CP0092), Canine PF4 kit, ABclonal, Woburn, MA.

^4^Cell Free DNA, SYTOX Green Nucleic Acid Stain (SYTOX Intensity, AU), Theromo Fisher Scientific, Waltham, MA.

^5^0.9% Sodium Chloride Inj, USP, Hospira, INC, Lake Forest, IL.

^6^SigmaPlot for Windows, Version 11, Systat Software.

^7^Albumin (Human) 25%, albumin 25, Kendrion Biopharma, Inc Fort Lee, NJ.

## Abbreviations

aPC: activated protein C
cfDNA: cell free DNA
IV: Intravenous
ODSH: 2-O, 3-O desulfated heparin
PF4: Platelet factor 4
TM: Thrombomodulin.

## References

[1] Singer M Deutschman CS Seymour CW Shankar-Hari M Annane D Bauer M. The third international consensus definitions for sepsis and septic shock (Sepsis-3). JAMA. (2016) 315:801–10. 10.1001/jama.2016.0287 26903338 PMC4968574

[2] Fryer A Huang YC Rao G Jacoby D Mancilla E Whorton R. Selective O-Desulfation Produces Nonanticoagulant Heparin that Retains Pharmacological Activity in the Lung. J Pharmacol Exp Ther. (1997) 282:208–19. 9223556

[3] Wang Y Luo L Braun OÖ Westman J Madhi R Herwald H. Neutrophil extracellular trap-microparticle complexes enhance thrombin generation via the intrinsic pathway of coagulation in mice. Sci Rep. (2018) 8:4020. 10.1038/s41598-018-22156-5 29507382 PMC5838234

[4] Camicia G Pozner R de Larrañaga G. Neutrophil extracellular traps in sepsis. Shock. (2014) 42:286–94. 10.1097/SHK.0000000000000221 25004062

[5] Kowalska MA Zhao G Zhai L David G III Marcus S Krishnaswamy S. Modulation of protein c activation by histones, platelet factor 4, and heparinoids. Arterioscler Thromb Vasc Biol. (2014) 34:120–6 10.1161/ATVBAHA.113.302236 24177324

[6] Joglekar MV Diez PM Marcus S Qi R Espinasse B Wiesner MR. Disruption of PF4/H multimolecular complex formation with a minimally anticoagulant heparin (ODSH). Thromb Haemost. (2012) 107:717–25. 10.1160/TH11-11-0795 22318669 PMC4441624

[7] Buchanan SA Kennedy TP MacArthur RB Meyerhoff ME. Titrimetric method for determination of O-desulfated heparin in physiological samples using protamine-sensitive membrane electrode as endpoint detector. Anal Biochem. (2005) 346:241–5. 10.1016/j.ab.2005.08.035 16213460

[8] Hayes G Mathews K Doig G Kruth S Boston S Nykamp S. The acute patient physiologic and laboratory evaluation (APPLE) score: a severity of illness stratification system for hospitalized dogs. J Vet Intern Med. (2010) 24:1034–47. 10.1111/j.1939-1676.2010.0552.x 20629945

[9] Gollomp K Sarkar A Harikumar S Seeholzer SH Arepally GM Hudock K. Fc-modified HIT-like monoclonal antibody as a novel treatment for sepsis. Blood. (2020) 135:743–54. 10.1182/blood.2019002329 31722003 PMC7059515

[10] Moreau D Vesin A Garrouste-Orgeas M de Lassence A Zahar JR Adrie C. Platelet count decline: an early prognostic marker in critically ill patients with prolonged ICU stays. Chest. (2007) 131:1735–41. 10.1378/chest.06-2233 17475637

[11] Llewellyn EA Todd JM Sharkey LC Rendahl A. A pilot study evaluating the prognostic utility of platelet indices in dogs with septic peritonitis. J Vet Emerg Crit Care. (2017) 27:569–78. 10.1111/vec.12628 28749085

[12] Letendre J Goggs R. Measurement of plasma cell-free DNA concentrations in dogs with sepsis, trauma, and neoplasia. J Vet Emerg Crit Care. (2017) 27:307–14. 10.1111/vec.12592 28295988

